# A Quality-of-Life Evaluation Study Assessing Health-Related Quality of Life in Patients Receiving Medicinal Cannabis (the QUEST Initiative): Protocol for a Longitudinal Observational Study

**DOI:** 10.2196/32327

**Published:** 2021-11-24

**Authors:** Margaret-Ann Tait, Daniel S J Costa, Rachel Campbell, Richard Norman, Stephan Schug, Claudia Rutherford

**Affiliations:** 1 School of Psychology, Faculty of Science The University of Sydney Sydney Australia; 2 School of Population Health Curtin University Perth Australia; 3 Medical School University of Western Australia Perth Australia

**Keywords:** medicinal cannabis, patient-reported outcomes, quality of life, chronic pain, pain management, mental health, depression, anxiety, cannabis oil

## Abstract

**Background:**

Evidence supports several countries introducing legislation to allow cannabis-based medicine as an adjunctive treatment for the symptomatic relief of chronic pain, chemotherapy-induced nausea, spasticity in multiple sclerosis (MS), epileptic seizures, depression, and anxiety. However, clinical trial participants do not represent the entire spectrum of disease and health status seen in patients currently accessing medicinal cannabis in practice.

**Objective:**

This study aims to collect real-world data to evaluate health-related quality of life in patients prescribed medicinal cannabis oil and describe any differences over time, from before starting therapy to after 3 and 12 months of therapy.

**Methods:**

Adult patients newly prescribed medicinal cannabis oil by authorized prescribers and under the Special Access Schemes across Australia will be screened for eligibility and invited to participate. A sample size of 2142 is required, with a 3-month follow-up. All participants will complete the EuroQol 5-Dimension; European Organization for Research and Treatment of Cancer Quality of Life Questionnaire-30; Depression, Anxiety, and Stress Scale-21; Patients’ Global Impression of Change; Patient-Reported Outcomes Measurement Information System (PROMIS) Short Form (SF) version 1.0: Sleep Disturbance 8b; and PROMIS SF Fatigue 13a questionnaires. Patients with chronic pain conditions will also complete the PROMIS SF version 1.0: Pain Intensity 3a and PROMIS SF version 1.0: Pain Interference 8a. Patients with movement disorders will also complete Quality of Life in Neurological Disorders (Neuro-QoL) SF version 1.0: Upper Extremity Function (Fine Motor and Activities of Daily Living) and if chorea is indicated, the Neuro-QoL SF version 2.0: Huntington’s Disease health-related Quality of LIFE-Chorea 6a. All questionnaires will be administered at baseline, 2 weeks (titration), monthly up to 3 months, and then every 2 months up to 1 year.

**Results:**

Recruitment commenced in November 2020. By June 2021, 1095 patients were screened for the study by 69 physicians in centers across 6 Australian states: Australian Capital Territory, New South Wales, Queensland, South Australia, Victoria, and Western Australia. Of the patients screened, 833 (39% of the target sample size) provided consent and completed baseline questionnaires. Results are expected to be published in 2022. Results of this study will show whether patient-reported outcomes improve in patients accessing prescribed medicinal cannabis from baseline to 3 months and whether any changes are maintained over a 12-month period. This study will also identify differences in improvements in patient-reported outcomes among patients with different chronic conditions (eg, chronic pain, MS, epilepsy, Parkinson disease, or cancer).

**Conclusions:**

This protocol contains detailed methods that will be used across multiple sites in Australia. The findings from this study have the potential to be integral to treatment assessment and recommendations for patients with chronic pain and other health indicators for accessing medicinal cannabis.

**Trial Registration:**

Australian New Zealand Clinical Trials Registry: ANZCTRN12621000063819; https://www.anzctr.org.au/Trial/Registration/TrialReview.aspx?id=380807&isReview=true

**International Registered Report Identifier (IRRID):**

DERR1-10.2196/32327

## Introduction

### Medicinal Cannabis

With the first accounts of cannabis being used as medicine dating back to China in 2600 BC, its use for medicinal purposes has been recorded on nearly every continent throughout history [1]. Many countries criminalized the consumption of cannabis in the 1900s, consequently limiting the potential therapeutic benefits and research into medicinal use. However, the identification of cannabinoids, cannabidiol, and Δ9-tetrahydrocannabinol as analgesics in 1940 refocused attention on using cannabis-based medicine as an adjunctive treatment for the symptomatic relief of chronic pain [1]. The last 2 decades have seen an increase in medicinal cannabis research, particularly in response to growing concerns about the misuse and adverse events associated with opioids [2], including increased risk of endocrinopathy, bowel dysfunction, cognitive decline, hospitalization, and death from overdose [3]. Research to date has provided sufficient evidence for several countries to introduce legislation allowing its use for medicinal purposes. These policies help to avoid the potential risk of cannabis abuse by self-medicating [4] and enable appropriate monitoring of possible adverse drug-drug interactions [5]. In 2020, there were approximately 400,000 medicinal cannabis patient registrations in Canada [6], more than 60,000 in Germany [7], and more than 25,000 in Australia [8].

Evidence from randomized controlled trials indicates that medicinal cannabis can reduce chronic pain [9-12], neuropathic pain [13], cancer pain [14], chemotherapy-induced nausea [15], spasticity in multiple sclerosis (MS) [16,17], epileptic seizures [18], depression [10,12], anxiety [12], improve sleep [19], and reduce opioid prescription numbers [20]. However, depression, anxiety, and sleep problems may be exasperated with formulations containing high ratios of Δ9-tetrahydrocannabinol [21], and for some health indications, using medicinal cannabis in a *real-world* setting may be confounded by drug-drug interactions [5]. This supports the need for oversight by health care professionals and further research collecting *real-world* evidence.

### Why Patient-Reported Outcomes Are Important

A patient-reported outcome (PRO) is any report coming directly from patients without interpretation by physicians or others about how the patient feels in relation to a health condition and its therapy [22]. PROs can include symptoms, aspects of functioning, multidimensional constructs such as health-related quality of life (HRQL), and perceptions of treatment. PROs are regarded as the gold standard for assessing pain [23] and are particularly important end points to include when assessing patients with chronic conditions where the primary aim is to palliate symptoms [24]. These patient reports were captured and quantified by PRO measures (PROMs) using validated and reliable standardized questionnaires that allow comparisons between treatment groups and within groups over time. The wide acceptance of PROM-based evidence by regulatory bodies is reflected in the Australian Commission on Safety and Quality in Health Care’s support of the use of PROMs to drive quality improvement [25] and the US Food and Drug Administration’s approval of PROs to support product labeling claims [26].

### Health Indications for Accessing Medicinal Cannabis

The Australian Therapeutic Goods Administration (TGA) currently approves Special Access Scheme (SAS) applications from health care providers who provide a clinical justification for prescribing medicinal cannabis where conventional therapies have failed [8]. There are no TGA-imposed restrictions on the types of health conditions; however, prescriptions are more commonly sought for chronic pain and spasticity from neurological conditions.

#### Chronic Pain

Chronic pain is a widespread health issue broadly defined as pain that lasts or recurs for more than 3 months [27] and is categorized as *chronic primary pain*—a disease in its own right such as nonspecific low-back pain—or as *chronic secondary pain* initiated as a symptom of an underlying disease, such as cancer-related pain or neuropathic pain [28]. In Australia, more than 15% of the adult population lives with persistent chronic pain or recurring pain lasting longer than 6 months [29]. In the United Kingdom, as many as 35% of adults experience some level of chronic pain lasting more than 3 months, with more than 10% reporting moderate to severely limiting chronic pain [30]. In 2016, the Centers for Disease Control and Prevention reported that 20% of US adults had chronic pain and 8% experienced chronic pain that severely interfered with daily functioning on most days or every day for a 6-month period [31].

Cancer-related pain is experienced by approximately one-third of patients with cancer at diagnosis and during treatment and by approximately three-fourths of patients with advanced-stage cancer. However, 10%-15% of the patients are nonresponsive to conventional pain therapy [32]. Residual tissue damage from cancer and cancer treatment often results in chronic pain in cancer survivors, which lasts many years after treatment [33].

Neuropathic pain, which is caused by a lesion or disease of the somatosensory nervous system [34], is experienced by approximately 8% of the population [35] and approximately 86% of the patients with MS [36]. Neuropathic pain is associated with higher rates of unemployment [35], poor physical, psychological, and social functioning, significantly impaired overall HRQL and sleep, and higher depression and anxiety than those with other chronic pain and those without pain [37]. Less than 35% of the patients with neuropathic pain respond to conventional therapy [38], whereas others often receive incomplete pain relief along with conventional treatment-related side effects [39].

The prevalence of chronic pain increases dramatically in patients receiving palliative care [40,41]. A study of patients in palliative care clinics in the United States found that most of them were admitted with unrelieved pain and that chronic pain assessment and management were inadequate [42]. In addition, one-third of the patients receiving palliative care who experienced pain were significantly more likely to also suffer from depression [41], report insufficient sleep [43], and were at a higher risk of opioid misuse [40]. Effective pain and symptom management aimed at reducing suffering and improving overall HRQL are the primary goals of palliative care.

#### Neurological and Movement Disorders

MS is a chronic inflammatory and demyelinating neurodegenerative disorder that often involves symptoms such as spasms, tremors, pain, fatigue, bladder dysfunction, cognitive impairment, depression, and impairments in swallowing, speech, vision, and balance [44]. In 2018, MS Research Australia reported an estimated 24,600 Australians with MS, and, on average, their overall HRQL was 31% less than the Australian population norm (measured by the health state utility valuation) [45]. As a currently incurable and often progressive condition, treatment and management largely focus on improving the quality of everyday life by relieving symptoms [44]. A systematic review of reviews conducted in 2018 on the effects of cannabidiol, Δ9-tetrahydrocannabinol, or cannabidiol and Δ9-tetrahydrocannabinol formulations in treating MS symptoms found sufficient evidence supporting cannabinoids in relieving both pain and spasticity symptoms [46].

Epilepsy is a chronic neurological disease characterized by 2 or more unprovoked seizures and affects approximately 50 million people worldwide [47]. High-quality evidence from randomized clinical trials suggests that cannabidiol reduces seizure frequency; however, further examination of PROs is needed to assess whether cannabidiol interacts with other antiseizure medications to produce unwanted side effects [48].

### Critical Gaps in Knowledge About HRQL in Patients Accessing Medicinal Cannabis

PRO assessment can assist health care professionals in monitoring treatment outcomes over time from patients’ perspectives. In 2020, there were no published results from a centralized PRO data collection for a large sample of patients accessing prescribed medical cannabis within Australia that covers all approved health indications using a comprehensive battery of PROMs. Participants studied under controlled clinical trial environments have not always been representative of the entire spectrum of disease and health status seen in people currently accessing medicinal cannabis in practice [49]. Therefore, although clinical trials provide evidence of the efficacy of medicinal cannabis, the true gauge of how effective it is in practice comes from real-world evidence from patients across all health conditions receiving prescribed medicinal cannabis [49]. Real-world data are needed to develop a scientific evidence base to inform regulation and policy making [7].

A scoping review that we conducted identified the following limitations in the current evidence on PROs for medicinal cannabis:

1. Very few studies have collected PRO data longitudinally, including baseline, maintenance, and long-term use data [4].
2. A large proportion of cannabinoid research was focused on pharmacokinetic, animal, and preclinical studies.
3. Many cannabinoid clinical studies include case studies [4] or have small sample sizes [50].
4. Early studies did not use the currently recommended individualized dosing titration paradigm of starting low and gradually escalating to achieve optimal effects [50].
5. Formulations studied may not reflect current commercially available cannabinoid products [11].
6. Many PROMs used have limited validity in the health conditions assessed [12].
7. Very few clinical studies used comprehensive pain assessments.

Therefore, the real-world collection of a comprehensive suite of PROs in a large sample of people across all health conditions, as approved by the TGA, accessing current formulations of prescribed medicinal cannabis in Australia is needed to enable clinically relevant assessment and provide ongoing evidence for decision-making both in practice and at a policy level.

### Objectives

The aim of this study is to evaluate PROs in patients who are prescribed medicinal cannabis by authorized prescribers and under the SAS across clinics within Australia. The findings from this study have the potential to be integral to treatment assessment and recommendations for chronic pain sufferers and other patients with health indicators for accessing medicinal cannabis.

#### Primary Objective

The primary objective of this study is to describe changes in the PROs (HRQL, pain, fatigue, sleep, anxiety, and depression) from baseline to 3 months for a large cohort receiving medicinal cannabis.

#### Secondary Objectives

The secondary objectives of this study are to describe changes in PROS (HRQL, pain, fatigue, sleep, anxiety, depression, and physical functioning) from baseline up to 12 months and to describe differences between patients accessing medicinal cannabis with different chronic health conditions, including, but not limited to, chronic pain, MS, epilepsy, Parkinson disease, and cancer.

#### Exploratory Objectives

The exploratory objectives of this study are to explore (1) which individuals are more likely to have lower symptom burden and greater HRQL, (2) associations among PROs, with the hypothesis that a high symptom burden is associated with poorer HRQL, and (3) associations between PROs and resource and medication use over time, with the hypothesis that lower symptom burden is associated with reduced health care–resource use and reduced use of opioids and other prescribed medications for managing symptoms.

#### Hypotheses

The study includes 3 hypotheses: (1) PROs will improve from baseline to 3 months in patients accessing medicinal cannabis, (2) improvements in PROs at 3 months will be maintained over a 12-month period, and (3) no differences in PROs will be observed between patients being treated for different conditions (eg, chronic pain, MS, epilepsy, Parkinson disease, or cancer).

## Methods

### Overview of Project Research Design

This is a multicenter prospective longitudinal cohort study of patients newly prescribed with medicinal cannabis in Australia by authorized prescribers and under the SAS. The study is registered in the Australian New Zealand Clinical Trials Registry (ACTRN12621000063819).

### Study Arrangements

To be eligible to participate in the study, participants must have already been identified as eligible to receive a medicinal cannabis product from an authorized prescriber or under the SAS category B pathway, with approval given by the Australian TGA. This means that a suitable health practitioner has seen and assessed their patient, adhering to relevant standards of good medical practice, and successfully applied to the TGA for access to the particular medicinal cannabis product for the patient. Little Green Pharma Ltd (LGP) is responsible for the manufacture and quality of the products following the TGA guidelines. The prescriber is responsible for the prescription of the product for the patient and seeking TGA approval either as an authorized prescriber or under the SAS-B scheme, including the patient’s informed consent for the product.

The University of Sydney researchers are responsible for the design of the cohort study and the data collection and analysis, as outlined in this protocol.

LGP is responsible for arrangements for delivery of the product, any subsidization arrangements, and the arrangements entered into with the participating sites and the physicians at these sites.

The prescriber is responsible for identifying the patients suitable for the study and obtaining consent to email them an invitation to participate in the study.

### Eligibility

The inclusion and exclusion criteria are provided in Textbox 1.

**Inclusion and exclusion criteria.**

**Inclusion criteria**

- Patient is an adult (aged ≥18 years).
- Patient has been identified as eligible to receive medicinal cannabis by a Therapeutic Goods Administration–approved authorized prescriber or through the Special Access Scheme (or equivalent in other countries and jurisdictions) and the physician has sought and obtained Therapeutic Goods Administration approval for the Little Green Pharma Ltd product for their patient.
- Patient is able to read and understand English.
- Patient is able to provide informed consent.
- Patient has not started any prescribed medicinal cannabis therapy in the last 4 weeks or started prescribed Little Green Pharma Ltd medicinal cannabis therapy within the last 2 days (we expect no therapeutic benefit within 2 days) and did not receive any prescribed medicinal cannabis therapy in the last 4 weeks.
- Patient has a life expectancy of >3 months.

**Exclusion criteria**

- Patient is unconscious or confused.
- Patient has cognitive impairment (eg, advanced Alzheimer disease).
- Patient is pregnant or breastfeeding.
- Patient is unable to read and write in English.
- Patient is denied access to medicinal cannabis under the relevant Special Access Scheme for their country of registration.

### Sample Size

#### Sample Size Considerations

Our aim is to recruit a large, broad, and representative sample of medicinal cannabis users. Therefore, we will invite every eligible patient treated at each participating center during a 12-month recruitment period. This large real-world cohort will enable several important analyses exploring differences in PROs between disease groups commonly treated with medicinal cannabis, as discussed in the *Objectives* section.

#### Minimum Sample Required for Primary Objective (Change Over Time)

Following the guidelines [51] for the European Organization for the Research and Treatment of Cancer Quality of Life Questionnaire-30 (EORTC QLQ-C30) [52] and allowing for a 20% loss to follow up, a baseline sample size of 2142 is required with a minimum follow-up of 3 months. This sample size provides 95% power to detect the smallest effect size threshold of 0.1 for the insomnia domain of the QLQ-C30, using a 2-tailed significance level of 1% [53].

### Recruitment and Consent Procedures

#### Screening

**Figure 1:**
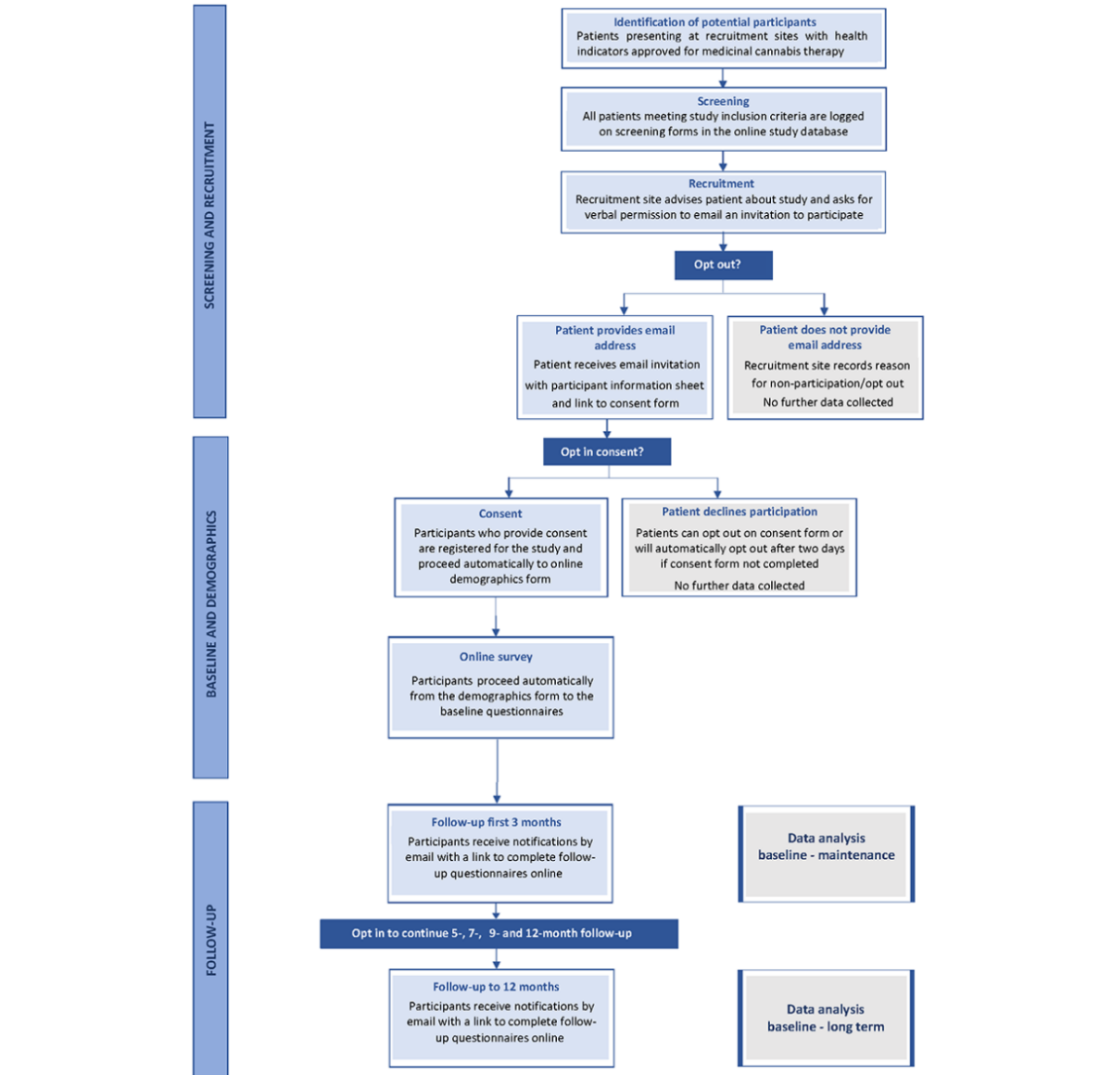
Patient recruitment and data collection process.

Recruitment will take place between November 2020 and November 2021, with the aim of including all eligible patients receiving medicinal cannabis at participating sites during the recruitment period. Figure 1 provides an overview of the patient recruitment and data collection procedures.

LGP, following the World Health Organization Guidelines on Good Agricultural and Collection Practices for Medicinal Plants and European Union-Goods Manufacturing Practices standards, provides its medicinal cannabis products in Australia under the SAS. As part of this process, LGP engages with medical cannabis–focused clinics, authorized prescribers, and other health care professionals prescribing LGP products and will be responsible for identifying recruitment sites for this study. Advertising and information relevant to the study will be disseminated via a dedicated study website and social media platforms. Content on the study website will inform potential participants and recruitment sites of the study objectives, terms, and conditions, including eligibility criteria, and who to contact for more information. Approved recruitment sites will receive study-specific training from the University of Sydney researchers regarding participant screening and recruitment.

Patients from participating centers who meet the eligibility criteria will be invited to participate in the study. Patients will be identified by physicians at recruitment sites and approached to participate by either the physician or site staff. A record of those identified as eligible and invited to participate will be logged in the web-based research database with the patient’s verbal consent. Physicians at recruitment sites will use a generic link to access the study database to create a new record for each patient screened.

The physician or staff at recruitment sites will ask if the patient agrees to have the Participant Information Statement and invitation sent to them through email. Patients will be informed that the process involves recruitment site staff entering the information, as per the *Registration and Clinical Data* section, into the research database, which then sends an invitation to them automatically. Participants will be informed that email addresses are stored within the Research Electronic Data Capture (REDCap) system and used solely for sending reminders to complete questionnaires at scheduled time points. REDCap [54] is a secure web-based application developed by Vanderbilt University that runs on the servers of the University of Sydney, ensuring that the data stay within the Sydney University data center.

All patients who provide their email address to receive an invitation for the study will receive their medicinal cannabis product at a standardized cost. As medicinal cannabis is still an unregistered product, it has been difficult to control the cost of the product; consequently, there has been considerable variability in what people pay for the product. LGP has partnered with several pharmacies across Australia to ensure that all participants taking part in this study will be charged the same amount for their product, eliminating variability in out-of-pocket costs and enabling a health economic evaluation.

As soon as an email address is entered in the web-based database, the patient will receive an email invitation with a link to the web-based forms for their record. The email will include the Participant Information Statement (Multimedia Appendix 1) to be considered before giving consent on the web. The Participant Information Statement contains detailed information about the rationale, design, and personal implications of the study. Patients will then provide their consent to join the study by checking the consent box before being able to proceed to the data collection forms or they can opt out of study participation at this point. They have as much time as they need to consider their participation. The patient’s right to refuse consent without giving reasons will be respected. If the patient does not respond to the invitation, 2 daily follow-up reminders will be sent via email, after which the system will automatically record the patient as having selected *opt out* without reason.

Participants will remain free to withdraw from the study at any time without giving reasons and without prejudicing any further treatment. Participants can withdraw responses from the study before the data have been analyzed; otherwise, they will be included.

#### Registration

When physicians at recruitment sites complete the web-based screening form, REDCap will automatically generate a number to be used as the participant’s study ID number for study registration.

#### Data Collection and Assessment

Study data will be recorded by physicians at recruitment sites on case report forms and by participants in the questionnaire booklets. These will be completed on the web through the University of Sydney research data capture system, REDCap [54].

Screening and registration and clinical data will be completed by the physician at the recruitment sites. The REDCap database will collect information only identifiable by REDCap-assigned study record ID numbers. Physicians at recruitment sites will maintain a record of participants’ study ID numbers for each screened and registered participant entered. Where required, data can be updated for individual participants by notifying the study project manager of the corresponding study ID numbers (eg, to record participant withdrawal). If 2 consecutive assessments are missed by participants, the study project manager will contact the physician at the recruitment site to determine the reasons for missed assessments, if known.

#### Registration and Clinical Data

The following patient screening details will be entered into the REDCap web-based registration form by physicians at recruitment sites: age, sex, country, email address, health indications for accessing medicinal cannabis, neuropathic pain screening using the short-form Douleur Neuropathique en 4 Questions [55,56] (if pain is selected as a health indication), duration of pain (if pain is recorded as a health indication), comorbidities, medicinal cannabis type and dosage, date and dose of any previously prescribed medicinal cannabis, recruitment physician ID, and reasons for declining study participation (if applicable).

#### Patient Consent and Demographics

Patients who provide email addresses will receive an email with a link to the patient consent and demographic form corresponding to their study record ID. Patient consent and demographic questions include the following: consent to participate (or opt out), reasons for declining study participation (if applicable), ethnicity and cultural background, education, living arrangements, marital status, height, weight, gender identity, work status, access to health services, any medication other than medicinal cannabis taken during the last 4 weeks for health indication, and previous history of cannabis use.

### PROM Administration

#### Administration

Baseline PROMs will be presented to participants on the web automatically after completing the demographic questions. Participants self-complete the questionnaires through the web-based platform at home, accessible on a computer or other device with an internet connection, depending on their preference. All the questionnaires will be administered in the same order. It is anticipated that completion of baseline questionnaires may take up to 30 minutes. Follow-up questionnaires may take approximately 25 minutes. We have estimated the time to complete the questionnaires (including demographic questions) based on 10-12 seconds per item [57].

#### Patient-Reported Outcome Measures

All participants will complete the following PROMs.

##### Generic HRQL

Generic HRQL will be assessed in all participants using the EuroQol 5-Dimension questionnaire (EQ-5D-5L). The EQ-5D-5L is a standardized measure of health status developed by the EuroQol Group to provide a simple, generic measure of health for clinical and economic appraisals [58]. EQ-5D-5L is designed for self-completion by respondents and consists of 5 items covering the dimensions of mobility, self-care, usual activities, pain or discomfort, and anxiety or depression. Ratings for each item range from 1 (no problem) to 5 (extreme problem), with a recall period of *today*. In addition to the 5 items, there is a visual numeric scale of global health rated on a scale of 0-100. The questionnaire has validated language translations suitable for use in Australia.

To make the EQ-5D-5L suitable for use in economic evaluations, health states were valued using a preference-elicitation method in the general population. Australian national values have been collected and subsequently modeled and will be used for economic analysis [59].

All participants will receive the EORTC QLQ-C30 core quality of life cancer questionnaire [52], which includes core domains of functioning, cancer-specific symptoms, fatigue, and general pain. The QLQ-C30 core questionnaire was designed to be used by any patient participating in a cancer clinical trial; however, it has also been used to evaluate HRQL in other health conditions [60-64], as well as in large general population samples in Europe, the United States, and Australia [65]. It is a 30-item questionnaire with a recall period of 1 week and contains 9 multi-item subscales and 6 single items. It incorporates 5 functional scales (physical, role, cognitive, emotional, and social functioning), 3 symptom scales (fatigue, pain, and nausea or vomiting), and a global health status and HRQL scale. The single items assess dyspnea, appetite loss, sleep disturbance, constipation, diarrhea, and perceived financial impact of disease and treatment. The ratings for each item range from 1 (not at all) to 4 (very much). The QLQ-C30 also produces a summary score of HRQL based on 13 scales [66].

The QLQ-C30 can be used for economic evaluation through the QLU-C10D [67], a health state classification system derived from the QLQ-C30 for which Australian utility weights have been established [68].

##### Overall Change in Health Status

Patients’ subjective rating of overall change in health status related to their primary health condition will be assessed using the Patients’ Global Impression of Change [69]. The Patients’ Global Impression of Change contains 1 item rated from 1 (very much improved) to 7 (very much worse). The recall period is *since beginning medicinal cannabis treatment*.

##### Anxiety and Depression

Anxiety, depression, and stress will be assessed in all participants with the validated 21-item short version of the Depression, Anxiety, and Stress Scale [70]. The Depression, Anxiety, and Stress Scale-21 includes 3 scales, each containing 7 items, assessing depression, anxiety, and stress. The depression scale assesses dysphoria, hopelessness, devaluation of life, self-deprecation, lack of interest or involvement, anhedonia, and inertia. The anxiety scale assesses autonomic arousal, skeletal muscle effects, situational anxiety, and subjective experience of anxious affect. The stress scale assesses difficulty relaxing, nervous arousal, and being easily upset or agitated, irritable or overreactive, and impatient. Ratings for each item range from 0 (not at all) to 3 (very much or most of the time) with a recall period of 1 week [71].

##### Sleep and Fatigue

Sleep quality will be assessed in all participants using the Patient-Reported Outcomes Measurement Information System (PROMIS) Short Form version 1.0: Sleep Disturbance 8b [72]. This measurement system is a universal, rather than disease-specific, 8 item assessment of sleep quality, sleep depth, and restoration associated with sleep. Items are rated from 1 (not at all) to 5 (very much so), with a recall period of 1 week.

Fatigue will be assessed in all participants using the PROMIS Fatigue 13a or the Functional Assessment of Chronic Illness Therapy Fatigue Scale [73]. The 13-item measure has been validated in the general population as well as in patients with cancer, anemia, and arthritis [74,75]. The scale consists of 2 domains: 5 items covering fatigue experience and 8 items assessing the impact of fatigue on daily activities. Ratings for each item range from 0 (not at all) to 4 (very much so), with a recall period of 1 week.

#### Conditional PROMs

The following questionnaires will only be administered to patients with identified conditions or health status.

##### Palliative Care

To reduce the burden on patients with a primary health indication of palliative care for advanced, symptomatic, incurable cancer with a life expectancy of a few months, they will receive the EORTC QLQ-C15-PAL instead of the QLQ-C30. It is a shorter, 15-item questionnaire that assesses the same outcomes as the QLQ-C30 questionnaire and is used extensively in the palliative care setting [76]. Palliative care participants will only complete the EQ-5D and QLQ-C15 questionnaires.

##### Pain

Participants with pain as a health indication in their baseline clinical data will complete additional pain-specific questionnaires (excluding those in palliative care):

Pain intensity will be assessed using the PROMIS Scale version 1.0: Pain Intensity 3a (PS-PI) [77]. The scale includes 3 items assessing pain intensity: 2 items cover pain at its worst, on average, over the last 1 week and 1 item about current pain. All items are rated from 1 (no pain) to 5 (very severe).

Pain interference will be assessed using the PROMIS Short Form version 1.0: Pain Interference 8a [78]. This measurement system contains 8 items measuring the degree to which pain interferes with physical, emotional, and social activities. Items are rated from 1 (not at all) to 5 (very much so), with a recall period of 1 week.

##### Motor Function

Participants with movement disorder, chorea, as a health indication will be assessed with the Quality of Life in Neurological Disorders Short Form version 2.0–Huntington’s Disease health-related Quality of LIFE-Chorea 6a [79]. This 6-item scale producing 1 score was developed for patients with Huntington disease and is appropriate for patients experiencing irregular, random, involuntary movements of varying amplitude affecting the face, trunk, and limbs. The domains cover the impact of movement disorders on physical activity and participation, with each item rated from 1 (never or not at all) to 5 (always or very much), with a recall period of 1 week.

Participants with movement disorders affecting the upper body as a health indication will be assessed using the Quality of Life in Neurological Disorders version 1.0: Upper Extremity Function (Fine Motor and Activities of Daily Living) Short Form [80]. This 8-item scale assesses the ability to perform various activities involving digital, manual, and reach-related functions, ranging from fine motor to self-care (activities of daily living) for patients with stroke, MS, amyotrophic lateral sclerosis, Parkinson disease, epilepsy, and muscular dystrophy. Items are rated from 1 (unable to do) to 5 (without any difficulty) and emphasize current capabilities; therefore, they do not use a recall period.

##### Work Status

For participants who indicate that they are working or would normally be working (ie, not retired or only studying), the impact of health on work performance will be assessed using the absenteeism and presenteeism questions of the World Health Organization’s Health and Work Performance Questionnaire [81]. The questionnaire contains 2 items covering absenteeism in the last 1 week and 2 items covering absenteeism in the last 4 weeks, rated in number of days. Presenteeism is covered by 3 items rated from 0 (worst performance) to 10 (top performance).

#### Follow-Up Data Collection

Participants will receive automatic reminders from the REDCap system to their email addresses at scheduled follow-up assessment time points (Table 1). Follow-up questionnaires can be completed using computers or mobile devices depending on their preference. Up to 2 email reminders to complete the follow-up questionnaires will be sent within the assessment time windows (Table 1). The following questions will be added to the front page of the follow-up questionnaires: current cannabis product and dose, any reduction in other medications taken for health indication because of using medicinal cannabis (including brand, strength, and dose), and work status.

**Table 1:** Patient-reported outcome assessment schedule.

PRO^a^ measure	Baseline	Titration^b^	1-month follow-up	2-month follow-up	3-month follow-up	5-month follow-up	7-month follow-up	9-month follow-up	12-month follow-up
		14-21 days after T0	4 weeks (and 3 days) after T1	8 weeks (and 7 days) after T1	13 weeks (and 7 days) after T1	21 weeks (and 7 days) after T1	30 weeks (and 7 days) after T1	39 weeks (and 7 days) after T1	52 weeks (and 14 days) after T1
	T0	T1	T2	T3	T4	T5	T6	T7	T8
EQ-5D^c^ questionnaire for measuring generic health status	✓	✓	✓	✓	✓	✓	✓	✓	✓
QLQ-C30^d^	✓	✓	✓	✓	✓	✓	✓	✓	✓
Depression, Anxiety, and Stress Scale-21	✓	✓	✓	✓	✓	✓	✓	✓	✓
PROMIS^e^ Short Form for Sleep Disturbance	✓	✓	✓	✓	✓	✓	✓	✓	✓
PROMIS Short Form for Fatigue-Fat	✓	✓	✓	✓	✓	✓	✓	✓	✓
Patients’ Global Impression of Change		✓	✓	✓	✓	✓	✓	✓	✓
World Health Organization Health and Work Performance Questionnaire: Absenteeism and Presenteeism questions	✓		✓		✓		✓		✓
PROMIS Scale for Pain Intensity^f^	✓	✓	✓	✓	✓	✓	✓	✓	✓
PROMIS Short Form for Pain Interference^f^	✓	✓	✓	✓	✓	✓	✓	✓	✓
Neuro-QoL^g^ Short Form for chorea^h^	✓	✓	✓	✓	✓	✓	✓	✓	✓
Neuro-QoL Upper Extremity Function Short Form^h^	✓	✓	✓	✓	✓	✓	✓	✓	✓
15-item version of QLQ-C30 for Palliative Care patients receiving palliative care^i^	✓	✓	✓	✓	✓				

^a^PRO: patient-reported outcome.

^b^The titration period is of approximately 2 weeks. As the EQ-5D assesses the current state (today), whereas the other patient-reported outcome questionnaires have a recall period of the past week, the time period is within 2-3 weeks to capture the end of titration across the questionnaires.

^c^EQ-5D: EuroQol 5-Dimension.

^d^QLQ-C30: Quality of Life Questionnaire, 30 items.

^e^PROMIS: Patient-Reported Outcomes Measurement Information System.

^f^Pain questionnaires will be only administered to participants with a health indication of pain.

^g^Neuro-QoL: Quality of Life in Neurological Disorders.

^h^Impact on Motor Function Questionnaire will be only administered to patients with a health indication of movement disorder.

^i^Participants in palliative care with life expectancy of a few months will only complete the EuroQol 5-Dimension and Quality of Life Questionnaire, 15 items.

#### PRO Assessment Time Points

Prospective assessment of newly prescribed patients before and after treatment is required to assess changes in PROs over time. PROs will be completed at baseline before starting medicinal cannabis, at 2-3 weeks after starting medicinal cannabis (end of titration period), and then at 1, 2, 3, 5, 7, 9, and 12 months after the titration period.

The acceptable PRO assessment time windows are indicated in Table 1.

### Analyses and Statistical Considerations

#### Analysis Set

All analyses will be performed using SPSS software (version 26.0; IBM Corp). Baseline demographic and clinical data will be summarized descriptively for all patients registered for the study. Categorical data will be presented as frequencies and percentages. For continuous scale data, mean, SD, median, 25th and 75th percentiles, and minimum and maximum scores will be presented.

PRO analyses will explore changes over time using mixed linear models. Subgroup analyses will compare differences in PROs between underlying conditions, dose, and duration of pain and over time using linear mixed models.

#### Statistical Considerations

A comprehensive PRO-specific statistical analysis plan will be produced by the study statistician. The key considerations include the following:

1. PRO questionnaire responses will be scored into PRO scales for outcome analysis according to standard scoring algorithms provided by the questionnaire developers and custodians.
2. Rates and reasons for missed PRO assessments will be summarized to assess likely missing data mechanisms against missing data assumptions of statistical modeling.
3. For each PRO, all participants with a score for that PRO at baseline and at least one time point after starting medicinal cannabis will be analyzed.
4. Linear mixed models will be used to compare groups in their PRO scores, adjusted for their PRO levels at baseline and with additional covariates such as duration of pain, previous cannabis use, use of other medications, and overall and prespecified subgroups.
5. If the scores are highly skewed, a suitable transformation will be sought to achieve normality.
6. As there are several PRO scales and time points and correlation among them is anticipated, statistical significance levels will be adjusted using an appropriate method [82].
7. The clinical significance of differences in the PRO questionnaires will be interpreted using existing guidelines (eg, QLQ-C30) [83], maintaining the overall type 1 error at 5% or less.

Missing items within the PRO questionnaires are not expected. This is because of the web-based administration platform alerting participants of missed items and the requirement to complete those items before progressing to the next page. Only those participants who complete at least 2 questionnaires (baseline and one other) will be included in the analysis. Single missed assessments will be imputed using the last value carried forward technique, that is, no change from that individual’s last assessment. Pattern mixture models will be used to impute scores for missed assessments based on recorded reasons [84].

#### Economic Evaluation

The economic evaluation will use collected data around pharmaceutical and other medical costs to explore the drivers of patient-level costs. As this study is not a comparative randomized trial, we are not proposing to conduct a formal economic evaluation, resulting in a cost per quality-adjusted life year. We will instead use baseline resource use as an indicator of typical care and contrast resource use throughout the study with baseline data. We will explore the relationship between HRQL and resource use across the cohort, which is potentially important information for future economic evaluation of medicinal cannabis.

## Results

Participant recruitment in Australia commenced on November 27, 2020. By June 4, 2021, 1095 patients were screened for the study by 69 physicians in centers across 6 Australian states: Australian Capital Territory, New South Wales, Queensland, South Australia, Victoria, and Western Australia. Of the 1095 patients screened, 833 (76.07%) participants provided consent, completed baseline questionnaires, and remained on the study. Baseline recruitment is expected to end in March 2022 when the target sample size of participants has completed the baseline questionnaires and a 3-month follow-up. The final results for the primary objective are expected to be published in 2022.

## Discussion

### Principal Findings

The results of this study will show whether PROs improve in patients accessing prescribed medicinal cannabis from baseline to 3 months and whether any changes are maintained over a 12-month period. This study will also identify whether there are differences in improvements in PROs among patients being treated for different conditions (eg, chronic pain, MS, epilepsy, Parkinson disease, or cancer).

### Conclusions

The findings from this study have the potential to be integral to treatment assessment and recommendations for chronic pain sufferers and other patients with health indicators for accessing medicinal cannabis.

## Appendix

Multimedia Appendix 1 QUEST participant information statement.

## Abbreviations

EORTC: European Organization for the Research and Treatment of Cancer
EQ-5D: EuroQol 5-Dimension
HRQL: health-related quality of life
LGP: Little Green Pharma Ltd
MS: multiple sclerosis
Neuro-QoL: Quality of Life in Neurological Disorders
PRO: patient-reported outcome
PROM: patient-reported outcome measure
PROMIS: Patient-Reported Outcomes Measurement Information System
QLQ-C15: Quality of Life Questionnaire, 15 items
QLQ-C30: Quality of Life Questionnaire, 30 items
REDCap: Research Electronic Data Capture
SAS: Special Access Scheme
TGA: Therapeutic Goods Administration
