# Supplementary material for: A Quality-of-Life Evaluation Study Assessing Health-Related Quality of Life in Patients Receiving Medicinal Cannabis (the QUEST Initiative): Protocol for a Longitudinal Observational Study
Source: JMIR Res Protoc. 2021 Nov 24;10(11):e32327. doi: 10.2196/32327 (PMC8663597; doi:10.2196/32327)
Supplement: Multimedia Appendix 1 [file resprot_v10i11e32327_app1.pdf]

THE UNIVERSITY OF SYDNEY

Quality of Life Office
School of Psychology
Faculty of Science

ABN 15 211 513 464

A/Prof CLAUDIA RUTHERFORD
Senior Research Fellow

Room 6.75
C39Z
The University of Sydney
NSW 2006 AUSTRALIA
Telephone: +61 2 8627 1583
Facsimile: +61 2 9036 5292
Email: claudia.rutherford@sydney.edu.au
Web: http://www.sydney.edu.au/

The QUEST initiative: QUality of life **E**valuation **St**udy
Assessing Health Related Quality of Life in patients receiving medicinal cannabis

PARTICIPANT INFORMATION STATEMENT

(1) What is this study about?

People accessing medicinal cannabis may have a range of conditions and symptoms that impact their quality of life. We would like to know how these conditions and symptoms impact quality of life and if this changes over time. The results from this study will help future patients with these conditions make decisions about their treatment.

You are invited to participate in this study because you are being prescribed medical cannabis. This Participant Information Statement tells you about the research study to help you decide if you want to take part. Please read this sheet carefully and ask questions about anything that you don't understand or want to know more about.

Participation in this research study is voluntary.

By giving your consent to take part in this study you are telling us that you:
✓ Understand what you have read.
✓ Agree to take part in the research study as outlined below.
✓ Agree to the use of your personal information as described.

(2) Who is running the study?

The study is being carried out by the following researchers:
- Dr Claudia Rutherford, Senior Research Fellow, School of Psychology, University of Sydney
- Ms Margaret-Ann Tait, Senior Research and Administration Officer, School of Psychology, University of Sydney

Margaret-Ann Tait is conducting this study for the degree of Doctor of Philosophy at The University of Sydney. This will take place under the supervision of Dr Claudia Rutherford.

This study is being funded by Little Green Pharma Ltd.

(3) What will the study involve for me?

Your doctor will identify if the study is suitable for you and enter some basic information about you into the study register. This includes your age, sex, health condition, and which medicinal cannabis product you are receiving. We will not collect any information that can identify you such as your name, physical address, or birth date.
You will be sent a link via email to join the study. When you open the link you will be asked if you consent to taking part.

If you decide you **do not** want to take part, select 'opt-out'. Your email address will be removed from the study. If you do not open the link, two reminders will be sent to you, after which you will automatically 'opt-out' and your email address will be removed from the study.

If you **do** wish to take part in the study, select 'yes'. After selecting 'yes' you can proceed to the first questionnaire. You will then receive emails to complete follow-up questionnaires when they are due.

The online questionnaire pack starts with some general information about you. For example, this information includes your living arrangements, whether you are employed, and what other medications you may be taking. This information is for research purposes only and will not be shared with your doctor.

You will then be asked about your quality of life and symptoms. Completing questionnaires involves choosing an answer to a set of questions. An example of a question that you might be asked is:

During the past week:<br>(select the number response that best applies to you)	Not at all	A little	Quite a bit	Very much
Have you had trouble sleeping?	1	2	3	4

Your completed questionnaire will be used only by researchers involved in the project and will be stored securely in Australia on the University of Sydney's servers.

You will complete the questionnaires on your own in English without interpretation from others.

(4) How much of my time will the study take?

You will complete questionnaires at the time you join the study, and then again at 2 weeks after starting medicinal cannabis therapy, monthly for 3 months, then every two months (i.e. 5, 7, and 9 months after starting medicinal cannabis therapy), and once again at 12 months. Questionnaires will take approximately 30 minutes to complete.

(5) Who can take part in the study?

You CAN take part in the study if:
- you are an adult (aged ≥18 years)
- you are able to read and understand English
- you are able to provide informed consent
- you have a life-expectancy of more than 3 months

- you have been identified as eligible to receive medicinal cannabis by a TGA approved Authorised Prescriber, or doctor using the special access scheme (SAS-B) (or equivalent in other countries and jurisdictions) and your prescribing doctor has TGA approval to prescribe Little Green Pharma products.
- you have:
    - not received any prescribed medicinal cannabis therapy in the previous 4 weeks, **or**
    - you have started prescribed medicinal cannabis therapy (Little Green Pharma product), within the previous 2 days and not received any prescribed medicinal cannabis therapy in the previous 4 weeks

You CANNOT take part in the study if you:

- are unconscious, confused or cognitively impaired
- are pregnant or breastfeeding
- are unable to speak, read and/or write in English
- are denied access to medicinal cannabis under the relevant Special Access Scheme in your country
- are unable to provide informed consent
- are currently receiving prescribed medicinal cannabis treatment for more than 2 days
- have received prescription medicinal cannabis within the last 4 weeks (excluding previous 2 days)

(6) Do I have to be in the study? Can I withdraw from the study once I've started?

Being in this study is completely voluntary and you do not have to take part. Your decision whether to participate will not affect your current or future relationship with the researchers or anyone else at the University of Sydney, and it will not affect your current or future treatment at the clinic you are having treatment or relationships with your doctor or treating team.

If you decide to take part in the study and then change your mind later, you can withdraw at any time by contacting the doctor who prescribed medicinal cannabis. If you withdraw from the study, you will no longer receive the study participant discount on the cost of your medicinal cannabis prescription.

Submitting your completed questionnaire is an indication of your consent to participate in the study. You can withdraw your responses from the study before the data has been analysed. After that point your data cannot be removed and will be included in the study.

(7) Are there any risks or costs associated with being in the study?

Aside from giving up your time, we do not expect that there will be any risks or costs associated with taking part in this study.

(8) Are there any benefits associated with being in the study?

Little Green Pharma have agreed to standardise the cost of their products across Australia and UK so that all patients taking part in this study will be charged the same discounted rate. The cost of medicinal cannabis products for participants will be set at **AUD150.00 per unit (bottle)**, plus delivery fee of $15.00. If you withdraw from the study, you may not receive the study participant discount on the cost of your medicinal cannabis prescription.

(9) What will happen to information about me that is collected during the study?

By providing your consent, you agree to us collecting personal information about you for the purposes of this research study. Personal and health information may include your age, health conditions, and the treatments that you have received. Your information will only be used for the purposes outlined in this Participant Information Statement, unless you consent otherwise.

All study information will be collected via the University of Sydney research data capture system, REDCap, a secure web application that runs on The University of Sydney's servers, ensuring data stays within the Sydney University data centre. Information will be stored electronically on the Research Data Store (RDS), a secure, password-protected server at the University of Sydney for at least 5 years, only accessible by the researchers, Dr Rutherford and Ms Tait. Any information that may identify you (such as email addresses) will be kept strictly confidential, except as required by law. Study findings may be published, but no individual participant will be identified in these publications.

We intend to give the information from this project to other researchers so that they can use it in their projects. Before we do so, we will take out all the identifying information so that the people we give it to won't know whose information it is. They won't know that you participated in the project and they won't be able to link you to any of the information you provided.

(10) Can I tell other people about the study?

Yes, you are welcome to tell other people about the study.

(11) What if I would like further information about the study?

If you would like more information about the study, please contact Margaret-Ann Tait at margaret-ann.tait@sydney.edu.au or phone +61 2 8627 1558.

(12) Will I be told the results of the study?

You can tell us that you wish to receive feedback by ticking the relevant box on the consent form. After the study is finished, you will be emailed a one page summary of the overall results.

(13) What if I have a complaint or any concerns about the study?

Research involving humans in Australia is reviewed by an independent group of people called a Human Research Ethics Committee (HREC). The ethical aspects of this study have been approved by the HREC of the University of Sydney [Project number: 2020/589]. As part of this process, we have agreed to carry out the study according to the *National Statement on Ethical Conduct in Human Research (2007).* This statement has been developed to protect people who agree to take part in research studies.

If you are concerned about the way this study is being conducted or you wish to make a complaint to someone independent from the study, please contact the university using the details below. Please quote the study title and protocol number.

The Manager, Ethics Administration, University of Sydney:

- **Telephone:** +61 2 8627 8176
- **Email:** human.ethics@sydney.edu.au
- **Fax:** +61 2 8627 8177 (Facsimile)

This information sheet is for you to keep
